# Detection of Permethrin pesticide using silver nano-dendrites SERS on optical fibre fabricated by laser-assisted photochemical method

**DOI:** 10.1038/s41598-019-49077-1

**Published:** 2019-08-29

**Authors:** Thanh Binh Pham, Thi Hong Cam Hoang, Van Hai Pham, Van Chuc Nguyen, Thuy Van Nguyen, Duc Chinh Vu, Van Hoi Pham, Huy Bui

**Affiliations:** 1 Institute of Materials Science, Vietnam Academy of Science and Technology, 18 Hoang Quoc Viet, Cau Giay, 100000 Hanoi Vietnam; 2 University of Science and Technology of Hanoi, Vietnam Academy of Science and Technology, 18 Hoang Quoc Viet, Cau Giay, 100000 Hanoi Vietnam; 3 Hanoi National University of Education, 136 Xuan Thuy, Cau Giay, 100000 Hanoi Vietnam

**Keywords:** Diode lasers, Environmental monitoring

## Abstract

Permethrin, 3-Phenoxybenzyl (1 RS)-cis,trans-3-(2,2-dichlorovinyl)- 2,2-dimethylcyclopropanecarboxylate, has a wide range of applications like insecticide, insect repellent and prevents mosquito-borne diseases, such as dengue fever and malaria in tropical areas. In this work, we develop a prominent monitoring method for the detection of permethrin pesticide using surface-enhanced Raman scattering (SERS) optical fibre substrates. The novel SERS-active optical fibre substrates were grown and deposited silver (Ag) nano-dendrites on the end of multi-mode fibre core by laser-assisted photochemical method. The characteristic of the Ag-nanostructures could be controlled by the experimental conditions, namely, laser illumination time. Ag nanoparticles optical fibre substrates and Ag nano-dendrites optical fibre substrates were prepared with laser illumination time of 3 min and 8 min, respectively. The achieved SERS-activity optical fibre substrates were tested with Rhodamine 6G aqueous solutions. We demonstrate that the SERS activity coupled with Ag nano-dendrites optical fibre substrate has higher Raman enhancement factor due to the creation of many of hot-spots for amplifying Raman signals. Besides, the stability and reproducibility of the Ag nano-dendrites optical fibre substrate were also evaluated with stored time of 1000 hours and relative standard deviation of less than 3%. The Ag nano-dendrite optical fibre substrate was selected for detection of permethrin pesticide in the concentration range of 0.1 ppm–20 ppm with limit of quantification (LOQ) of 0.1 ppm and calculated limit of detection (LOD) of 0.0035 ppm, proving its great potential for direct, rapid detection and monitoring of permethrin.

## Introduction

Pesticides are exploited in agricultural production intended for increasing the yield, as well as improving the quality of crops. The pyrethroid family pesticide including permethrin, cypermethrin, deltamethrin, λ-cyhalothrin, which is of natural origin by mixture of various complex ester-shaped separated from the flowers of the daisy, has been widely used in agriculture as insecticide, acaricide, and insect repellent. In which, permethrin does not present any notable genotoxicity or immunotoxicity in humans and farm animals, but is classified by the US Environmental Protection Agency (EPA) as a likely human carcinogen, and is listed as a “restricted use” substance^1^. However, the pesticide misuse leads to serious environmental pollution and public concern since it could pose potential risks to human health. Therefore, the accurate, rapid and reliable detection of pesticides will prevent potential harmful effects on the environment and human health. Normally, pesticides are detected by various laboratory based techniques, such as chromatography, gas chromatography-mass spectrometry, high-performance liquid chromatography, atomic fluorescence spectrometry, liquid chromatography-tandem mass spectrometry^2–4^. These techniques have high sensitivity and accuracy but they rely on expensive instruments and require professionally trained personnel to treat samples and operate instruments in centralized laboratories. Hence, it is essential to develop various new generation sensors, such as colorimetric, electrochemical, acoustic, field-effect transistor (FET) devices^5,6^, surface-enhanced Raman scattering (SERS)^7–9^, and optical fiber sensors^10–12^. The bio-chemical sensors based on optical fibers are rapidly developed because of their advantages such as low signal attenuation, compact size, small sample volume, high flexibility, immunity from interference of electromagnetic fields, and remote sensing possibility. The SERS is a powerful spectroscopic technique for ultrasensitive and selective bio-chemical detection^13–15^ due to its capability of providing “fingerprint” of information of molecular structures in low concentrations. The enhancement mechanism of SERS has been explained by the highly local enhanced electromagnetic field on the noble metal surface due to the excitation of localized surface Plasmon resonances and enhanced chemical interaction between the adsorbate and noble metal nanoparticles. The regions with a highly enhanced local electromagnetic field are often called ‘hot spots’, which play a decisive role in the enhancement of the Raman signal. The Raman signal enhancement highly depends on the arrangement and morphology of the noble metal surface. The SERS on metal surface roughening or aggregation (such as flowers-like or dendrites) has demonstrated large enhancement factors of SERS activity^16–19^. The SERS substrates with many hot-spots and uniform distribution of nanostructures on the surface will have a large Raman enhancement and high repetition of measurement. The metal dendritic nanostructures are structures with many hot-spots which are formed from the tips and the sharp edges of the trunk and branches of the nano-dendrites, and the narrow gap between the branches^20,21^. The Ag nanostructure is considered to be one of the most excellent candidates for SERS application due to its highly desired plasmonic properties, low cost and easy fabrication/synthesis. The dendritic Ag nanostructure has been of interest and usually fabricated by electrochemical deposition method to increase high density hot-spots for enhanced SERS activity^22,23^. Photochemical approach has been used to synthesize Ag nanostructures suspended in solution by directly exposing on growth solution^19,24–26^. Recently, several studies combining SERS activity with optical fibre for directional development SERS optical fibre probe of biochemical compact optical fibre sensors using in outdoor fields have been recognized^27–29^. Ag nanoparticles have been modified on the silanized surface which was created by functionalizing SERS optical fibre probe^30–32^ or the Ag nanoparticles have been synthesized on the fiber taper surface through chemical deposition method^33^.

In this paper, we propose a novel SERS-active on optical fibre substrates with synthesized silver nano-dendrites structure by a facile and low-cost laser-assisted photochemical method. The Ag nanostructures were directly grown and immobilized on the multi-mode fibre ends by irradiation of green laser beam from a mixed silver ions solution, and occurred only in the main laser-irradiated part. Rhodamine 6G aqueous solutions were employed as a probe to characterize the enhancement, stability and uniformity of the achieved SERS substrates with Ag nanostructures on the optical fibre ends. It is found that the Raman enhancement factor of the Ag nano-dendrites SERS optical fibre substrate was increased. This SERS optical fibre substrate also exhibited good stability and reproducibility with an average relative standard deviation of less than 3%. The proposed Ag nano-dendrite optical fibre substrates were applied in detection of permethrin pesticide in the concentration range of 0.1 ppm–20 ppm with LOQ and LOD of 0.1 ppm and 0.0035 ppm, respectively. The goal of this study was to investigate the feasibility of this proposed SERS optical fibre substrate and prove its great potential for chemical and environmental compact optical fibre sensors.

## Materials and Methods

### Materials

Optical multimode fibre with core/cladding diameter of 62.5/125 µm and NA of 0.22 (Thorlabs, USA) was chosen in the experiments. Silver nitrate (AgNO_3_, 99.5% purity), tri-sodium citrate dihydrate (C_6_H_5_Na_3_O_7_.2H_2_O, greater than 99% purity) and sodium borohydride (NaBH_4_) were purchased from Fisher Scientific UK, Merck KGaA (Germany) and Kanto Chemical. Co. Inc. (Tokyo, Japan), respectively. Permethrin (C_21_H_20_Cl_2_O_3_, its purest commercially available grade) and Rhodamine 6G (R6G) were supplied by Sigma-Aldrich (Switzerland). All the aqueous solutions were prepared with ultrapure water (greater than 18 MΩ).

### Instruments

All the SERS optical fibre substrates were prepared by assisted-diode laser with emission wavelength of 532 nm and power of 500 mW (Laserlands, China). The scanning electron microscope (SEM) images and Energy dispersive X-ray spectroscopy (EDX) were obtained by a field-emission SEM (FE-SEM Hitachi S-4800) with the acceleration voltage of 5 kV. All measured samples were prepared by using BDL-pipetter 6601 (Becton Dickinson Labware, USA). The Raman scattering measurements were performed by a Raman Microscope system (Horiba Scientific LabRAM HR Evolution) with confocal microscope connected to the objective lens of 10x, 60x, 100x and an excitation wavelength of 532 nm.

### Preparation of the Ag nanostructure SERS optical fibre substrates

**Figure 1:**
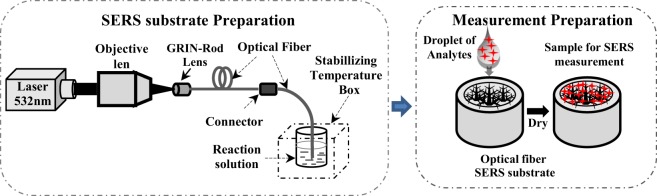
Schematic diagram of the SERS optical fiber substrates preparation.

The schematic diagram of the experimental setup to prepare the Ag nanostructure SERS on the end of the optical fibre with assisted-diode laser is illustrated in Fig. 1. First, the growth solution was prepared by mixing an optimal molar concentration of aqueous 0.2 ml of AgNO_3_ (0.01 M) and 0.2 ml of tri-sodium citrate (0.3 M) with 19.56 ml of ultrapure water in a clean tumbler. After rapid stirring for 3 min, a freshly prepared solution of 0.04 ml of sodium borohydride solution (0.01 mM) was added dropwise to the mixture under vigorous stirring for 5 min, and after that it was kept in the dark at room temperature for further use. Second, the polymer-coating layer of the multi-mode optical fibres were stripped along 25 mm from the end, the end of fibre was carefully cleaved so that the surface of fibre-end was perpendicular to the fibre axis, and the samples were cleaned by ethanol. Then, the growth and immobilisation of Ag nanostructure on the end of the optical fibre cores were performed with assisted-diode laser propagated through the optical fibre. It was focused onto one end of multimode optical fibre (62.5/125 µm) glued to the grin-rod via an objective lens of 10x (NA = 0.22), the other end of multimode optical fibre had a standard fibre connector for collecting laser beam to fibre sample. The optical fibre sample was fixed on a homemade fibre holder in which the position of the optical fibre sample can be finely adjusted and was dipped into the prepared Ag-chemical solution, and then the laser beam was propagated into the solution via the optical fibre. Characterization of Ag nanostructures formed on the end of fibre core depended on the exposure time of diode laser. During the Ag nanostructure preparation process, the reaction solution was contained in a reactor vessel with stable temperature of 20 °C by using Peltier thermoelectric cooler to avoid the effect of the temperature on the growth of Ag nanostructure. When the Ag nanostructure preparation process was finished, the nano Ag-coated optical fibre substrate was taken out and carefully rinsed with deionized water and then dried with pure nitrogen gas stream. The Ag nanostructures coated on the optical fibre substrate remained stable in the further exposure with 532 nm laser beam in air and/or in liquid environment without Ag^+^ ions.

### Measured sample preparation

In general, R6G is used to test the stability of SERS substrate^34,35^ so in our research, we first prepare the R6G test solution in order to verify the stability of the fabricated SERS substrates before analyzing the performance of permethrin. A test aqueous solution of R6G (10^−5^ M, 10^−7^ M and 10^−8^ M) was prepared by being diluted in ultrapure water from stock solution of R6G (10^−2^ M). 13 mL of 1000 ppm stock analytic solution was prepared by dissolving 10 mg Permethrin in 12.62 ml high-purity methanol. The following series of standard solution concentrations of 20 ppm, 10 ppm, 5 ppm, 1 ppm, 0.5 ppm and 0.1 ppm were formed by diluting it in ultrapure water due to the limited solution of the permethrin analyte in water.

Then droplets of 1 µL of the R6G-diluted solutions were dropped on the SERS optical fibre substrate and naturally dried in air before SERS detection so that R6G molecules could be absorbed onto the Ag nanostructures in order to test the stability of SERS substrate. Similarly, permethrin solution was analysed with this approach to investigate the monitoring ability by fabricated SERS optical fibre substrate.

The Raman scattering measurements were performed at room temperature by a Raman spectrometer system (Horiba Scientific LabRAM HR Evolution) using the objective lens of 100x, excitation wavelength of 532 nm and laser power of 3 mW. The SERS optical fibre substrate was fixed perpendicularly to the objective lens by a fibre holder, and focused on by exciting laser beam. The Raman spectra were obtained with acquisition time of 30 s and two accumulations. All Raman spectral data were background-subtracted by the spectral acquisition software (LabSpec 6.0 from Horiba Jobin Yvon) and averaged from randomly four measured point on the optical fibre substrate.

## Results and Discussion

**Figure 2:**
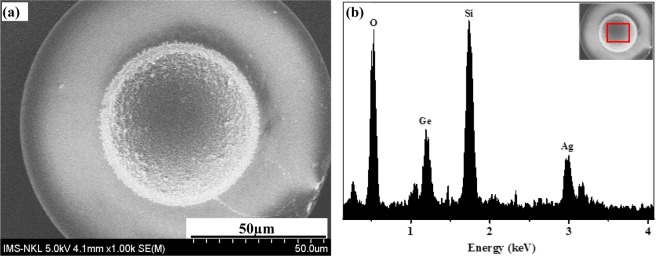
SEM image (**a**) and EDX spectrum (**b**) of silver nanostructure SERS-active on optical fibre substrate, the inset shows EDX measured zone.

**Figure 3:**
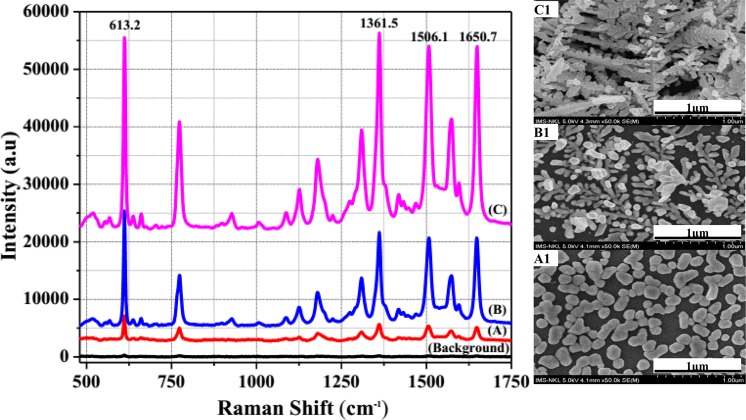
SERS spectra of R6G solution (10^−5^ M) recorded for SERS substrate with growth Ag nano-pebbles (curve A), Ag nano-aggregates (curve B) and Ag nano-dendrites (curve C) on the end of optical fibre core, and the (A1), (B1) and (C1) SEM images, respectively.

The morphology characterization of the fabricated silver nanostructure SERS optical fibre substrates were exhibited by FE-SEM images as shown in Figs 2 and 3. The silver nanostructures were uniformly distributed and confined within the core area of the end of multimode fibre of 62.5/125 µm as clearly depicted in Fig. 2(a). The components of the silver nanostructure SERS optical fibre substrate were analysed by investigating the EDX spectrum measured in the marked region of the inset as displayed in Fig. 2(b), in which the sharp peaks as O, Ge and Si corresponding to the components of typical optical fibre with Ge doped into SiO_2_ substrate and the sharp peak Ag definitively demonstrated Ag nanostructures growth after assisted-laser. The fundamental mechanism of growth of silver nanostructures on the end of optical fibre core could be interpreted by the fact that spherical silver seed particles were first formed through the reduction of silver nitrate by sodium borohydride in the presence of tri-sodium citrate, after under illumination of laser beam with optical power density of 78 W/cm^2^, suspended spherical silver seeds in growth solution were promptly driven toward the region where the optical field was more intense and were stuck and formed onto the surface of the end optical fibre core because of the optical gradient force near the surface of the end optical fibre core by assisted-laser beam. The silver nanostructures were grown because more silver ions were strongly reduced and deposited onto silver seeds formed previously due to the surface plasmon resonance effect of the silver seed^36–39^. The evolution of the growth of silver nanostructures can be clearly observed with different exposure time as demonstrated by SERS spectra shown in Fig. 3(with curves A, B, C) and the SEM images depicted in Fig. 3(A1, B1, C1 correspond to these curves). The silver nano-pebbles structure from 70 to 180 nm was uniformly formed onto the surface of the end optical fibre core under illumination of laser beam within short exposure time of 3 min, as depicted in Fig. 3(A1). After exposing for 7 min, silver nano-aggregates were generated and the silver short branches were developed with the size from 90 to 120 nm as shown in Fig. 3(B1). Since the exposure time was increased to 8 min, the silver nano-dendrites structure was formed with higher density and the branches were enlarged to the size of around 110–160 nm as represented in Fig. 3(C1). To investigate the SERS ability of the silver nanostructures optical fibre substrates prepared at different exposure time of laser, the SERS spectra of the R6G solution of 10^−5^M modified onto the prepared silver nanostructures optical fibre substrates were recorded in Fig. 3 (curves A, B, C). Some of the strong bands of R6G-adsorbed silver nanostructure were clearly observed with the feature characteristic peaks at 613.2 cm^−1^ assigned to the C-C-C ring in-plane, and 1361.5 cm^−1^, 1506.1 cm^−1^ and 1650.7 cm^−1^ corresponding to the symmetric modes of C-C in-plane stretching vibrations^40^. We could also observe that the SERS signal intensity of R6G was greatly enhanced by increasing exposure time of silver nanostructure growth process. The characteristic Raman signal intensity of R6G-adsorbed silver nano-dendrites structure optical fibre substrate is much higher than that of silver nano-pebbles structure optical fibre substrate at 613.2 cm^−1^, 1361.5 cm^−1^, 1506.1 cm^−1^ and 1650.7 cm^−1^ with 7.25, 11.44, 11.87, and 13.1 fold, respectively. To quantitatively estimate the Ag nanostructure-induced enhancement, we could calculate the relative enhancement factor (REF) according to different exposure time. The REF of SERS optical fibre substrate strongly depends on the SERS conditions, such as morphology of substrate, analytic, excitation wavelength, etc., and can be calculated by the formula^17,41^:

1 $${\rm{REF}}=\frac{{I}_{S{\rm{ER}}S}\times {C}_{R}}{{I}_{R}\times {C}_{SERS}}$$

where I_SERS_ is the SERS intensity of R6G adsorbed on an Ag nanostructure optical fibre substrate and I_R_ represents the normal Raman intensity (non-SERS) of R6G on the optical fibre probe without Ag nanostructure. C_SERS_ is the concentration of R6G (10^−5^ M) in SERS spectrum, C_R_ is the concentration of R6G (1 M) in normal Raman spectrum as depicted in Fig. 3 (Background line). The R6G with the concentration of 1 M was tested on the optical probe without silver nanostructure, the corresponding intensity at 613.2 cm^−1^ is 321 in arbitrary unit (a.u.). The enhanced intensity of R6G with the concentration of 10^−5^ M on the silver nano-dendrites structure on the optical fibre substrate is 33392.6 in a.u. The calculated values of REF of characteristic Raman signals of R6G-adsorbed silver nano-pebbles structure optical fibre substrate, silver nano-aggregates structure and silver nano-dendrites structure optical fibre substrate at 613.2 cm^−1^, 1361.5 cm^−1^, 1506.1 cm^−1^ and 1650.7 cm^−1^ were summarized in Table 1. We can clearly observe that the REF for characteristic Raman signals of R6G molecule with concentration of 10^−5^M increased as the process of developing of Ag nanostructures following to the exposed time increasing. The longer exposed time generates exotic silver morphology structures. The REF value was highest in the case of the silver nano-dendrites structure optical fibre substrate prepared at exposed time of 8 min. The great REF of SERS signals onto this Ag nano-dendrites structure optical fibre substrate could be postulated for two SERS enhancement mechanisms which are the electromagnetic enhancement and the chemical enhancement. Chemical enhancement involves charge transfer between the SERS substrate and the detected molecules. Consequently, the high performance SERS signal intensity of this Ag dendrites structure optical fibre probes could be primarily assigned to an electromagnetic enhancement effect. This effect is generated from the light induced localized surface plasmon resonance (LSPR). The short distances among dendritic branches, the large curvature regions between the trunk and branches and the fractal features of Ag dendrites nanostructure may allow the formation of many SERS ‘hot-spots’ where the optical field intensity is much higher than that at other sites. The Ag nano-dendrites structure optical fibre substrate could provide a number of highly active hot-spots that may promote giant electromagnetic enhancement thus obtaining a higher enhancement factor value. In the case of silver nano-pebbles, SERS ‘hot spots’ are only located between the nano-pebbles while the branches of silver nano-aggregates are short and the density is low so their REF values are not so large.

**Table 1:** Values of REF of characteristic Raman signals of R6G-adsorbed silver nanostructures optical fibre substrates.

Silver nanostructure on optical fibre substrates	Raman shift (cm^−1^)			
	613.2	1361.5	1506.1	1650.7
Silver nano-pebbles structure	1.43 × 10^6^	1.28 × 10^6^	0.93 × 10^6^	0.84 × 10^6^
Silver nano-aggregates structure	6.27 × 10^6^	7.04 × 10^6^	5.3 × 10^6^	5.31 × 10^6^
Silver nano-dendrites structure	1.04 × 10^7^	1.47 × 10^7^	1.1 × 10^7^	1.1 × 10^7^

**Figure 4:**
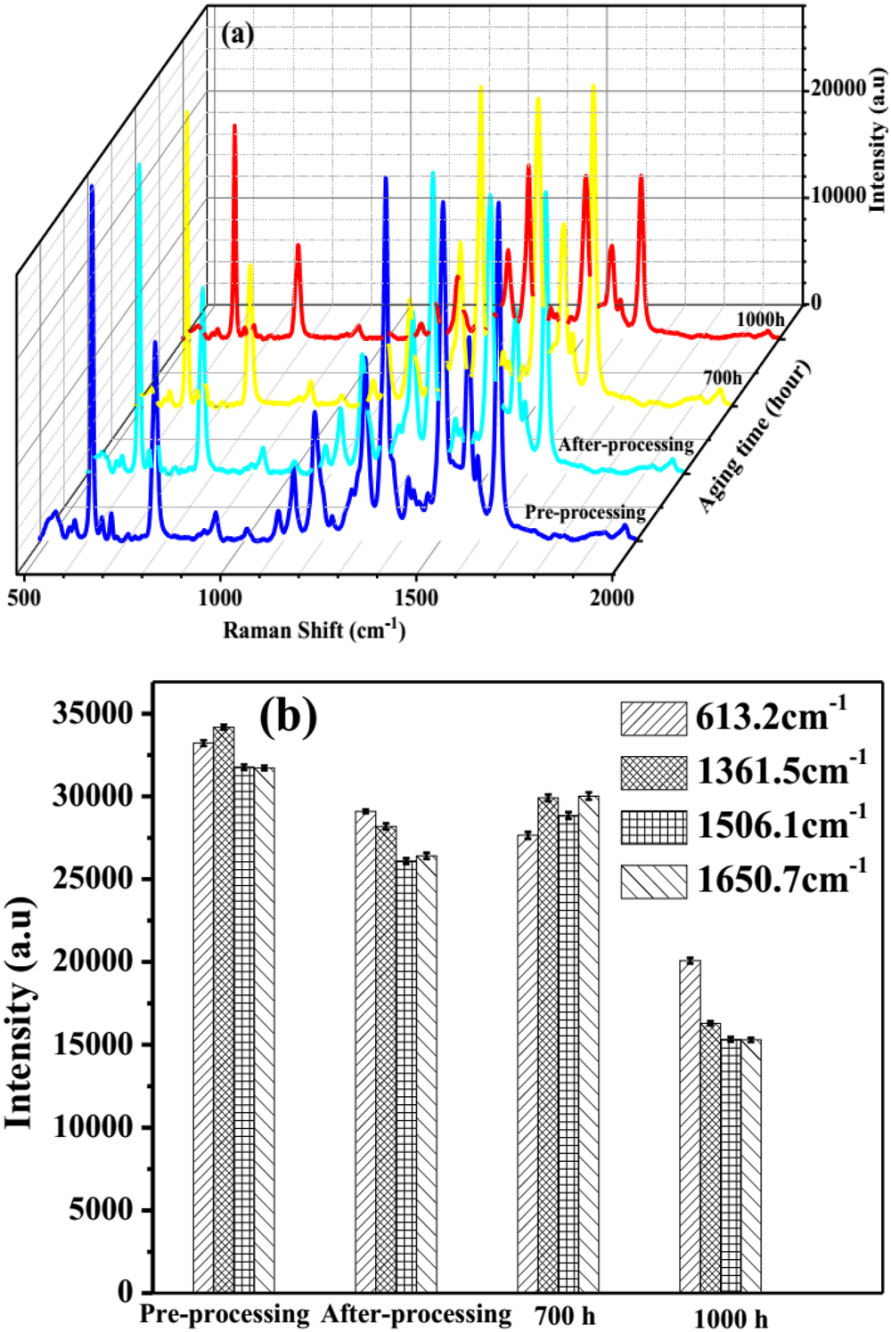
(**a**) SERS spectra of 10^−5^ M R6G modified on the Ag nano-dendrite structure optical fibre substrate corresponding pre-processing substrate, after-processing substrate, and substrates stored for 700 h and 1000 h. The Raman signals intensities at 613.2 cm^−1^, 1361.5 cm^−1^, 1506.1 cm^−1^ and 1650.7 cm^−1^ decreased after processing surface of Ag nano-dendrite SERS optical fibre substrate and stored substrate time of 700 h, 1000 h (**b**).

**Figure 5:**
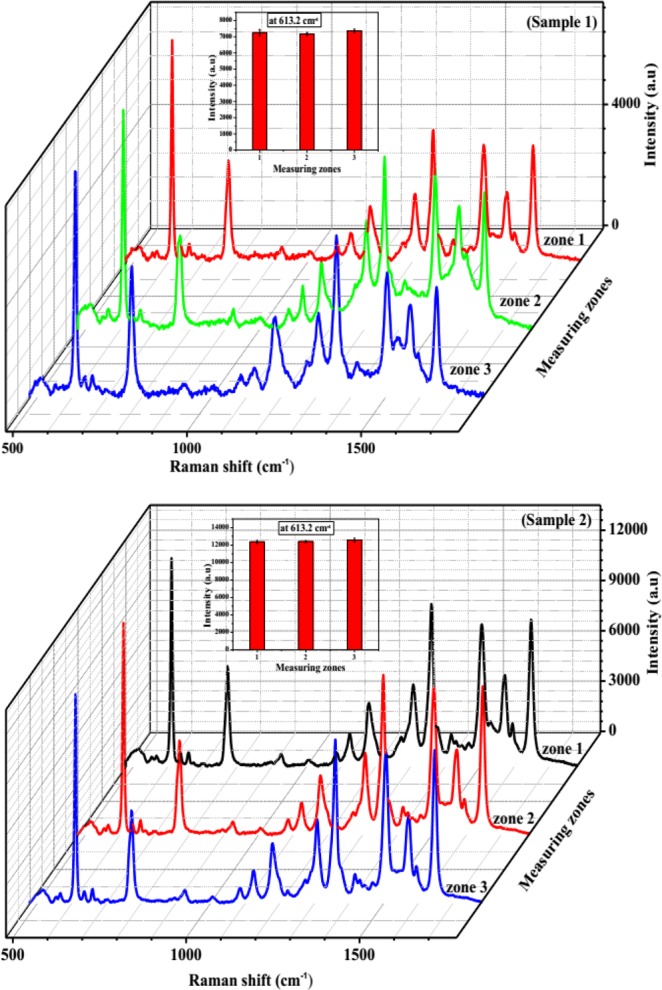
SERS spectra of 10^−8^ M and 10^−7^ M R6G modified from three different zones of two of the Ag nano-dendrite structure optical fibre substrates with the relative standard deviation of the Raman signals at 613.2 cm^−1^ as shown in inset show in (sample1) and (sample2), respectively.

**Figure 6:**
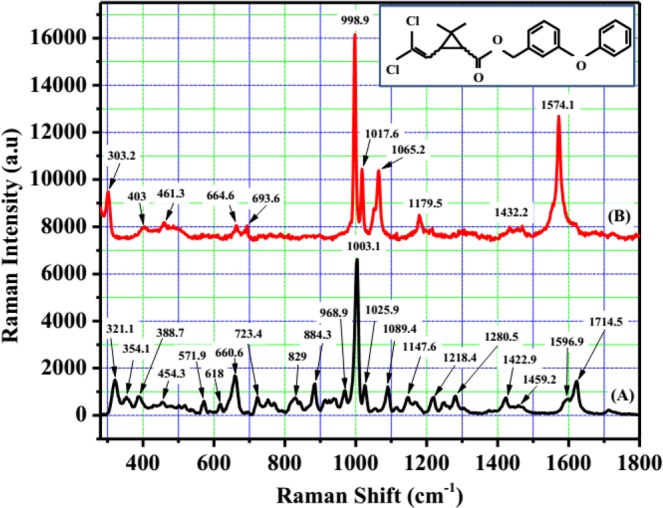
Normal Raman spectrum of permethrin solid on optical fibre substrate (**A**) and SERS spectrum of 10 ppm permethrin solution recorded for Ag nano-dendrite SERS optical fibre substrate (**B**), the inset shows molecular structure of permethrin.

**Figure 7:**
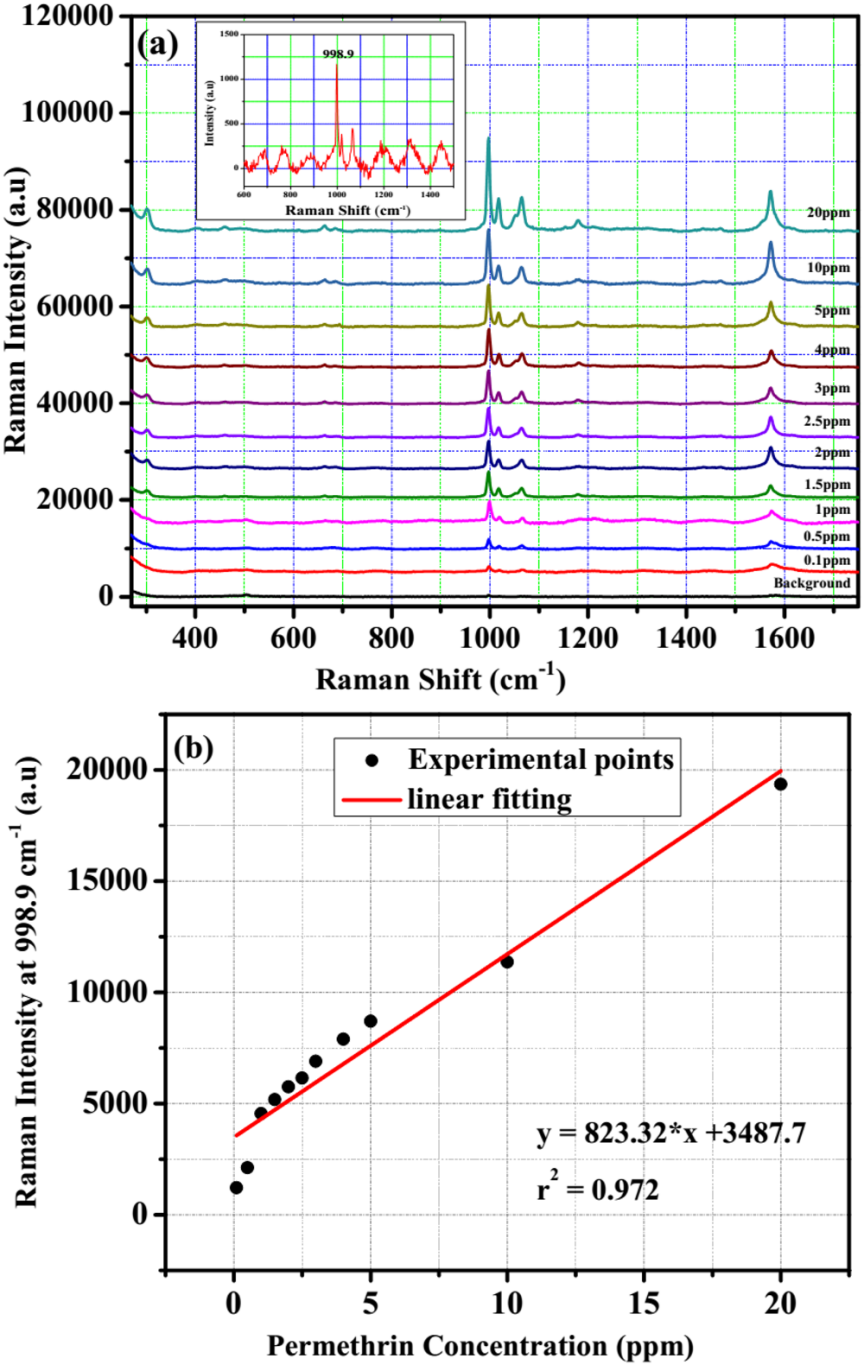
SERS spectra of permethrin solutions with different concentrations such as 0.1 ppm, 0.5 ppm, 1 ppm, 1.5 ppm, 2 ppm, 2.5 ppm, 3 ppm, 4 ppm, 5 ppm, 10 ppm and 20 ppm using Ag nano-dendrite SERS optical fibre substrate (**a**). The dependence of the vibrational band intensity at 998.9 cm^−1^ on the permethrin concentration (**b**), and the inset shows fitting of the linear region of the permethrin concentration of 0.1 ppm–5 ppm.

Beside the high enhancement and sensitivity of SERS substrate, stability and reproducibility are also important parameters to evaluate the quality of SERS substrate. The stability of Ag nano-dendrites structure optical fibre substrate can be improved by limiting the oxidation of its surface, which was implemented by exposing the 532 nm laser beam in air immediately after finishing preparation. Figure 4(a) exhibits the Raman spectra of 10^−5^M R6G modified on the Ag nano-dendrites structure optical fibre substrate corresponding pre-processing substrate, after-processing substrate, and substrates stored for 700 h and 1000 h. Figure 4(b) shows that the intensity of Raman signals at 613.2 cm^−1^, 1361.5 cm^−1^, 1506.1 cm^−1^ and 1650.7 cm^−1^ decreased after processing the surface of SERS optical fibre substrate of about 14.6%, 15%, 13.5%, and 11.1%, respectively, and then the intensity of Raman signals stabilized during stored substrate time of 700 h. It was demonstrated that the silver nano-dendrites structure was firmly attached on the surface of the end optical fibre substrate. The intensity of Raman signals at 613.2 cm^−1^, 1361.5 cm^−1^, 1506.1 cm^−1^ and 1650.7 cm^−1^ decreased after storing time of 1000 h of about 29.2%, 43.9%, 44.2%, and 45.8%, respectively, owing to the oxidation of silver. Figure 5 presents Raman spectra of R6G modified from three different zones of the Ag nano-dendrites structure optical fibre substrate, the spectra of each zone is analysed by four mapping runs. The intensity of Raman signals on that three different zones display no shift and are nearly similar as depicted in Fig. 5 (Sample 1 with R6G of 10^−8^ M) and Fig. 5 (Sample 2 with R6G of 10^−7^ M). The relative standard deviation of the Raman signals at 613.2 cm^−1^ was calculated to be about 3% as shown in inset of Fig. 5 which proves the reproducibility of the measurements. Therefore, these results illustrate that the SERS substrate with Ag nano-dendrites structures on the end of fibre core prepared by assisted-laser possesses optimum SERS activity at an exposure time of 8 min could be a good candidate for an improved SERS optical fibre probe. The silver nano-dendrite SERS-active on optical fibre substrates were selected for detection of permethrin with molecular formula of C_21_H_20_Cl_2_O_3_ and molecular structure shown in the inset of Fig. 6. The Raman spectrum of permethrin solid with 99.5% purity on optical fibre substrate without Ag nanostructure and the SERS spectrum of 10 ppm permethrin solution on the optical fibre substrate with silver nano-dendrites are clearly shown in Fig. 6. The primary characteristic Raman peaks of permethrin are marked in the spectra corresponding to assignments of the bands enumerated in Table 2^42^. The strong Raman bands of permethrin-adsorbed Ag nano-dendrites are clearly observed with the feature characteristic peaks at around 303.2 cm^−1^ associated with the scissoring vibration of C-O-C, the strongest peak at 998.9 cm^−1^ assigned to the benzene ring breathing mode, 1017.6 cm^−1^ and 1179.5 cm^−1^ corresponding to the stretching vibration mode of C-O, 1065.2 cm^−1^ assigned to the scissoring vibration mode of C-H on benzene ring, and 1574.1 cm^−1^ corresponding to the symmetric modes of C-C in-plane stretching vibrations, which are enhanced and shifted a few cm against the feature characteristic peaks in Raman spectrum of permethrin solid on optical fibre substrate without Ag nanostructure. The interaction of permethrin solution with silver nano-dendrites optical fibre substrate might account for these small shifts of the feature characteristic peaks in the SERS spectrum. The SERS spectra of permethrin solution with different concentrations of 20 ppm, 10 ppm, 5 ppm, 4 ppm, 3 ppm, 2.5 ppm, 2 ppm, 1.5 ppm, 1 ppm, 0.5 ppm and 0.1 ppm dispersed onto the silver nano-dendrites optical fibre substrates produced by the same process are exhibited in Fig. 7(a). It can be seen that the intensity of the SERS signal increases as the concentration of permethrin solution increases. The strongest Raman peak at 998.9 cm^−1^ assigned to the benzene ring breathing mode are clearly observed, so this Raman peak could be applied for the quantitative SERS detection of permethrin solution. Figure 7(b) represents the dependence of the intensity of the strongest SERS peak at 998.9 cm^−1^ from permethrin on the concentration of permethrin solution. Linear regression at the strongest SERS peak at 998.9 cm^−1^ is given as I = 823.32*C + 3487.7, as shown in the inset of Fig. 7(b), which proves the applicability of the silver nano-dendrites optical fibre substrate for quantitative analysis of permethrin pesticide. The LOD is defined as LOD = 3 × SD/slope, where SD is the standard deviation of the noise^43^, and the determined value is 0.0035 ppm. Comparing with LOD of permethrin on silver nanofilm deposited on glass chip^44^, the LOD of the silver nano-dendrites optical fibre substrate is far less. According to the food hygiene regulation of Vietnam Ministry of public health (No: 50/2016/TT-BYT), the maximum residue level (MRL) of permethrin residue in green or black tea is 20 ppm. Hence, this SERS optical fibre is a perfect candidate for the application in permethrin pesticide detection.

**Table 2:** The primary characteristic Raman peaks of permethrin corresponding to their assignments^42^.

NR Raman bands (cm^−1^)	SERS bands (cm^−1^)	Assignments
321.1	303.2	δ (C-O-C)
354.1	—	τ (C-O-C-C)
388.7	—	δ (C-C-C)
454.3	403	γ (=C-Cl)
571.9	461.3	In-plane deformation mode of benzene ring
618	—	υ_sym_ (C-Cl)
660.6	664.6	υ_sym_ (C-Cl)
723.4	693.6	In-plane deformation mode of benzene ring
829	—	In-plane deformation mode of cp
884.3	—	υ (C-C)
968.9	—	γ (C-H)_cp_
1003.1	998.9	breath of benzene ring
1025.9	1017.6	υ (C-O)
1089.4	1065.2	δ (C-H)_benzene_
1147.6		δ (C-H)_benzene_
—	1179.5	
1218.4	—	υ (C-O)
1280.5	—	breath of cp
1422.9	—	δ (C-H)_cp_
1459.2	1432.2	δ_asym_ (CH_3_)
1596.9	1574.1	υ (C=O)
1714.5	—	υ (C=O)

Note: δ: scissoring; τ: torsion; γ: out-of-plane bending; υ: stretching; sym: symmetric; cp: cyclopropyl; asym: asymmetric.

## Conclusion

In conclusion, we have successfully detected permethrin pesticide by preparing a novel silver nano-dendrites structure SERS-active on optical fibre substrate by a facile and low-cost laser-assisted photochemical method. The Ag nano-dendrites SERS optical fibre substrates showed an extremely higher Raman enhancement factor than that of nano-pebbles structure and also exhibited good stability and reproducibility with an average relative standard deviation of less than 3%. The Ag nano-dendrite optical fibre substrates were applied in detection of permethrin pesticide in the concentration range of 0.1 ppm–20 ppm with LOQ of 0.1 ppm and calculated LOD of 0.0035 ppm. The Ag nanostructures were directly grown and immobilized on the multi-mode fibre ends by irradiation of green laser beam from a mixed solution contented silver ions, and occur only in the main laser-irradiated part were confirmed by SEM images. The surface of silver nano-dendrites structure SERS optical fibre substrate was prepared and treated simultaneously by the assisted-laser 532 nm via optical fiber. In this treatment, Rhodamine 6G aqueous solutions were employed as a probe to characterize the enhancement, stability and uniformity of the achieved SERS substrates with Ag nanostructures on the optical fibre. The developed SERS optical fibre substrates can contribute as a promising candidate for portative SERS equipment with direct, rapid, real-time and non-destructive detection of pesticides residue in the liquid environment in outdoor fields.
